# Nanoparticles and Colloidal Hydrogels of Chitosan–Caseinate Polyelectrolyte Complexes for Drug-Controlled Release Applications

**DOI:** 10.3390/ijms21165602

**Published:** 2020-08-05

**Authors:** Aastha Lall, Arnaud Kamdem Tamo, Ingo Doench, Laurent David, Paula Nunes de Oliveira, Christian Gorzelanny, Anayancy Osorio-Madrazo

**Affiliations:** 1 Laboratory for Sensors, Institute of Microsystems Engineering IMTEK, University of Freiburg, 79110 Freiburg, Germany; aastha.lall@imtek.uni-freiburg.de (A.L.); arnaud.kamdem@imtek.uni-freiburg.de (A.K.T.); ingo.doench@imtek.uni-freiburg.de (I.D.); 2 Freiburg Materials Research Center FMF, University of Freiburg, 79104 Freiburg, Germany; 3 Freiburg Center for Interactive Materials and Bioinspired Technologies FIT, University of Freiburg, 79110 Freiburg, Germany; 4 Laboratoire Ingénierie des Matériaux Polymères IMP, CNRS UMR 5223, University of Lyon, University Claude Bernard Lyon 1, CEDEX, 69622 Villeurbanne, France ; laurent.david@univ-lyon1.fr (L.D.); paulaoliveiraqmc@gmail.com (P.N.d.O.); 5 Experimental Dermatology, Department of Dermatology and Venereology, Medical University Hamburg-Eppendorf, 20246 Hamburg, Germany; c.gorzelanny@uke.de

**Keywords:** polymer nanoparticles, colloidal hydrogels, polyelectrolyte complex, chitosan, caseinate, drug-controlled release

## Abstract

Chitosan–caseinate nanoparticles were synthesized by polyelectrolyte complex (PEC) formation. Caseinate is an anionic micellar nanocolloid in aqueous solutions, which association with the polycationic chitosan yielded polyelectrolyte complexes with caseinate cores surrounded by a chitosan corona. The pre-structuration of caseinate micelles facilitates the formation of natural polyelectrolyte nanoparticles with good stability and sizes around 200 nm. Such natural nanoparticles can be loaded with molecules for applications in drug-controlled release. In the nanoparticles processing, parameters such as the chitosan degree of acetylation (DA) and molecular weight, order of addition of the polyelectrolytes chitosan (polycation) and caseinate (polyanion), and added weight ratio of polycation:polyanion were varied, which were shown to influence the structure of the polyelectrolyte association, the nanoparticle size and zeta potential. Attenuated total reflection-Fourier transform infrared (ATR-FTIR) analyses revealed the chemical structure of hydrogel colloidal systems consisting of nanoparticles that contain chitosan and caseinate. Transmission electron microscopy (TEM) allowed further characterization of the spherical morphology of the nanoparticles. Furtherly, insulin was chosen as a model drug to study the application of the nanoparticles as a safe biodegradable nanocarrier system for drug-controlled release. An insulin entrapment efficiency of 75% was achieved in the chitosan-caseinate nanoparticles.

## 1. Introduction

The application of nanomaterials is gaining increasing interest in the fields of medicine, healthcare and drug delivery, electrical and information technology sectors, energy systems, agricultural industry, etc. [1]. Nanomaterials can perform several functions, such as (i) the targeted and sustained administration of bioactive molecules, in order to improve the bioavailability of a drug or to reduce its toxicity thanks to targeted delivery to cells/tissues/organs; (ii) imaging to visualize damaged tissue, perform diagnosis, and/or follow a therapeutic treatment. Dispersions containing particles of dimensions commonly in the range from 5 to 500 nm, with a surrounding interfacial layer and dispersed in a continuous phase, are commonly distinguished as colloidal systems. Colloidal nanocarriers can be multifunctional. On a subcellular scale, they can be used for targeting a cell compartment to allow their imaging while releasing an active product, observing the distribution of the drug and local effect. As medical devices, the applications of these nanosystems is governed by a regulatory context, where proof of safety, performance, up-scalability, and process robustness at every step of the fabrication should be considered. Thus, there is the need to design safe nanomaterials with a sufficient lifetime for delayed drug administration or imaging applications, with sufficient clearance to avoid accumulation and associated toxicity, for example, in the liver or spleen.

In the healthcare sector, polymer nano-/microparticles are revolutionizing biomedicine [2,3]. They can substantially improve drug performance by providing better drug shielding, enhancing the specificity of the delivery, promoting its sustained release and tissue healing, regeneration, etc. [4,5,6].

Oral drug administration is the most suitable route as it eliminates pain and the possible risk of infections involved with the repetitive use of syringes. However, a suitable drug carrier system, which entraps and protects the drug from harsh pH and enzymatic environment of the gastrointestinal (GI) tract is necessary [7].

Biomacromolecules like polysaccharides, proteins, polyphenols, nucleic acids, among others, are nowadays of major interest for the most varied applications due to their high availability [8,9,10,11,12,13,14,15], biodegradability and biocompatibility [16,17]. Biopolymers can carry charges according to the environmental conditions to which they are exposed, like the pH of the physiological medium [16,17]. Specifically, macromolecules like polysaccharide and proteins can form polycations, polyanions or zwitterionic polymers. The association between a polyanion and a polycation yields a polyelectrolyte complex (PEC) [5]. The driving force for their formation is electrostatic in nature, but additional interactions like hydrogen bonding, dipole–dipole, and charge transfer also can contribute to their stabilization. Polyelectrolyte association should be controlled in order to obtain a robust processing. Charged macromolecule interactions in solution depend on many factors like the intrinsic nature of polyelectrolytes (chain arrangement, rigidity/flexibility, mobility, solubility), their molecular weight, charge density, and external factors, such as polyelectrolytes concentration, medium ionic strength, pH, temperature, etc. [18].

Chitosan (CHI) is a linear co-polysaccharide of D-glucosamine and N-acetyl-d-glucosamine units linked by β (1→4) glycosidic bonds. In weakly acidic aqueous media, CHI is a cationic polyelectrolyte due to the protonation of its amine groups [19,20,21,22]. The presence of this positive charge is key to achieving solubility in weakly acidic aqueous media, which in turn can be exploited to form films [23], fibers [24], hydrogels [16,17], and nano-/microparticles [25], and immobilize substances such as proteins [25] and peptides, with potential applications in cosmetics [26], food industry, biotechnology [25], biomedical devices for wound dressing, contact lens antibacterial treatments, and drug encapsulation [27,28], as it is nontoxic, biodegradable, and bacteriostatic [29]. Janes et al. [30] and Prabaharan et al. [31] showed the use of chitosan as nanoparticle carriers for several drug delivery systems. Numerous polyelectrolyte complexes (PECs) have been formed by chitosan and other molecules [32], such as dextran sulfate [33], acylated cruciferin [34], hyaluronic acid [35], pectin, and nucleic acids [36]. Chitosan based NPs can be synthesized via different methods, including spray drying, coating a layer of chitosan over synthesized micro/nanoparticles, ionic gelation, solvent evaporation, and PEC formation as reviewed by Zhao et al. [29] and Wu et al. [32].

Casein(s) are a group of proteins found in bovine milk. The caseins are amphiphilic phosphoproteins which can self-assemble into spherical micelles which are held together by colloidal calcium phosphate [37,38]. Casein is soluble at pH ranging from 6 to 7 which is above the isoelectric point of 4.6–4.8. A reduction in pH causes it to aggregate along with precipitation of calcium [39]. Specially, in the sodium caseinate dispersion system, a dissociation of the native micelles into sub-micelles is produced [40]. Some authors have studied nano and microparticles based on sodium caseinate. Sodium caseinate/gelatin particles were non-spherical in shape and had irregular surfaces [41], but calcium phosphate–PEG–casein particles stayed in the intestinal tract long enough for an efficient delivery of insulin, as they were muco-adhesive [42].

Regarding the properties of chitosan-based NPs, several structural and processing factors can affect the NP features, such as the chitosan (CHI) polymer molecular weight and degree of acetylation (DA) [43] and polyelectrolyte being used in excess during PEC formation [34]. Nanoparticles loaded with insulin, made of CHI and poly(glutamic acid) were shown to be effective in reducing blood sugar levels in diabetic rats after oral administration [43]. Nanoparticles prepared with chitosan (CHI) and dextran sulfate (DS) were also shown to have a high insulin association efficiency of 85% [28]. This was attributed to the presence, in insulin, of a six amino acids sequence capable of bearing a positive charge likely to bind DS, and a ten amino acids sequence that could bind chitosan [44]. Other CHI-based NPs based on this polyelectrolyte and tripolyphosphate (TPP) [45] had an insulin association of about 80% with improved intestinal absorption of insulin in vivo. Anal et al. [46] reported the preparation of chitosan–caseinate NPs of 250–300 nm size, in which studies the drug chloramphenicol was used to assess sustained-release, showing the suitability of the CHI–caseinate system as nanocarriers [47].

In this work, taking advantage of the rationale for the development of chitosan–caseinate nanoparticles as natural, non-toxic, and biocompatible colloidal systems on the one hand and insulin-loaded chitosan-based nanoparticles on the other, we investigated the development and structural properties of CHI–caseinate polyelectrolyte nanoparticles in which process the screening of major parameters like the CHI molecular weight and degree of acetylation as well as order of addition of the polyelectrolytes CHI and caseinate, and added polycation:polyanion weight ratio were investigated in a systematic way. Finally, CHI–caseinate nanocarrier systems obtained following different NP development approaches were loaded with insulin to evaluate the sustained drug release.

## 2. Results

### 2.1. Chitosans Macromolecular Structure and Thermal Properties

**Figure 1:**
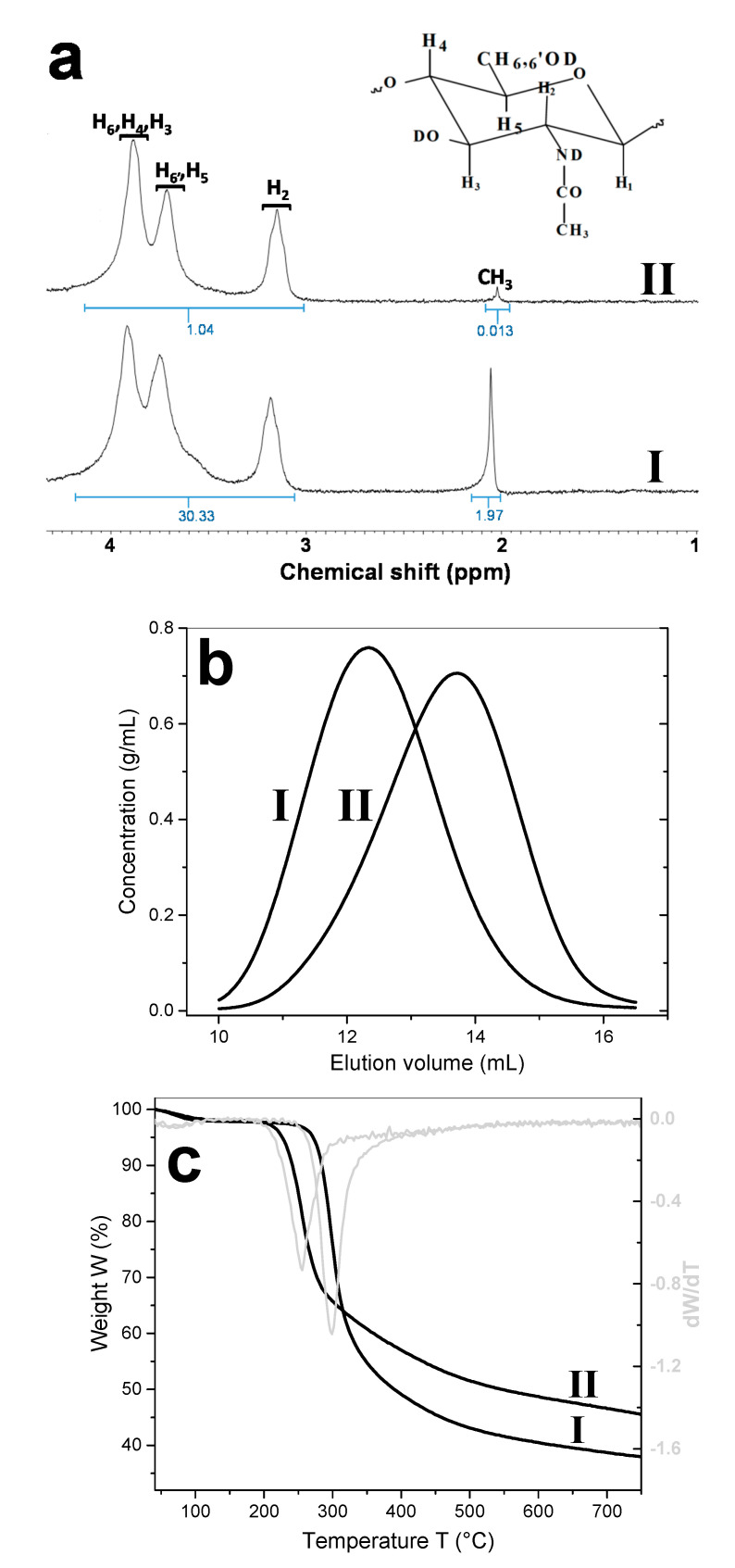
(**a**) ^1^H NMR spectra of: (I) the low molecular weight chitosan (LMW CHI (raw material)) and (II) the highly deacetylated low molecular weight chitosan (deacetylated-LMW CHI), with values of signals integrated intensities written in blue; (**b**) size exclusion chromatography (SEC) chromatograms of: (I) LMW CHI and (II) deacetylated-LMW CHI; (**c**) thermogravimetric analysis (TGA) of: (I) LMW CHI and (II) deacetylated-LMW CHI.

Three different chitosans were studied in this work. The ^1^H NMR nuclear magnetic resonance spectroscopy analysis of the chitosan (CHI) samples allowed estimating a degree of acetylation (DA) of: 3% for the starting high molecular weight HMW CHI supplied by Mahtani Chitosan, 13% for the commercial low molecular weight LMW CHI (Sigma Aldrich (Taufkirchen, Germany)); and 3% after performing almost complete deacetylation of the LMW CHI (Figure 1a) by using a freezing-pumping-thawing (FPT) process previous to the deacetylation reaction, as described below in Section 4.

Figure 1b shows the size exclusion chromatography (SEC) analyses, obtained by using a refractometry index RI detector, of the starting LMW CHI and after deacetylation (deacetylated-LMW CHI). The obtained chromatograms revealed that the initial molecular weight of the LMW CHI was slightly reduced after “full” deacetylation. The summary of the obtained values of number-average molecular weight (Mn), weight-average molecular weight (Mw), and polymer polydispersity index (PDI), along with the degree of acetylation (DA) of the different chitosans are shown in Table 1. Thus, the characterization of the different chitosans further allowed comparative studies to investigate the effect of CHI molecular weight and DA on the properties of the chitosan–caseinate polyelectrolyte systems considered in the nanoparticles processing.

The thermogravimetric analyses (TGA) of Figure 1c show that after a previous water evaporation weight loss step at around 100 °C, which allowed estimating a polymer natural hydration of around 2.5% (w/w) for the different chitosans, the TGA curves practically revealed one thermal degradation step. The highest rate of weight loss was observed at 300 °C for the LMW CHI of a higher degree of acetylation DA = 13%, and at 255 °C for the deacetylated-LMW CHI of DA = 3%. This reveals a minor reduction of the chitosan thermal stability under the achieved chitosan deacetylation [48].

### 2.2. Spectroscopic Study of Chitosan/Caseinate Interaction

The chitosan–caseinate interactions were firstly investigated by FTIR and Raman spectroscopy analyses of chitosan–caseinate casted films.

#### 2.2.1. Fourier-Transform Infrared Spectroscopy (FTIR)

**Figure 2:**
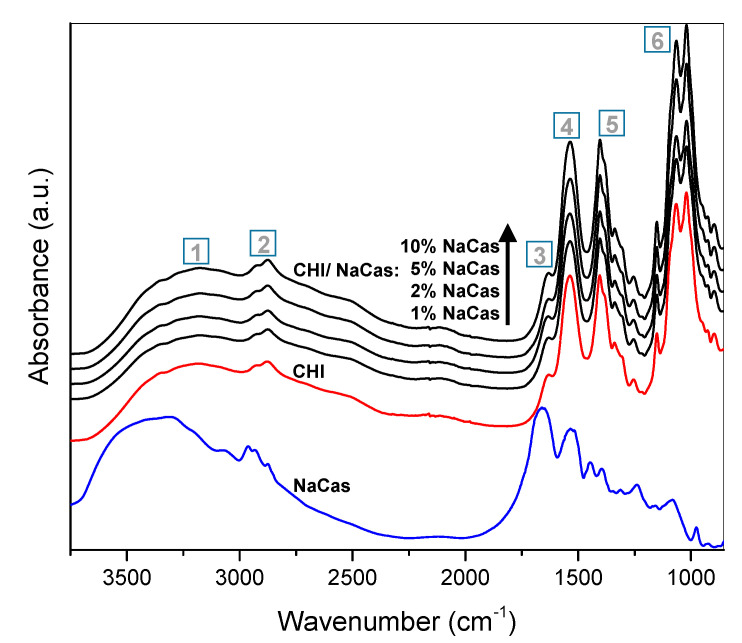
FTIR spectra of oven-dried HMW CHI chitosan/caseinate films prepared by varying the concentration of sodium caseinate (NaCas): 1%; 2%; 5% and 10% (w/w). The assignment of the bands regions 1, 2, 3, 4, 5, and 6 are discussed in detail above in the text in Section 2.2.1.

The FTIR spectra of the films of chitosan HMW CHI acetate alone and of chitosan/caseinate are displayed in Figure 2. The major identified regions are: (1) broad signal at wavenumber region 3180–3350 cm^−1^ for O-H and N-H stretching, where usually two broad bands of chitosan at 3393 and 3298 cm^−1^ overlap, attributed to the presence of the intra- and intermolecular H-bonds, respectively; (2) bands at 2920 and 2874 cm^−1^ are associated to C–H group asymmetric and symmetric stretching vibrations, respectively; (3) at 1630 cm^−1^ for C=O stretching associated to the amide I group and NH_3_^+^ asymmetric deformation, the carbonyl band of which is usually observed at ∼1670 cm^−1^ for chitosan in its free amine form but shifts to a lower wavenumber when sodium caseinate is added to chitosan acetate to form a CHI–caseinate complex film, or just when acetic acid is added to chitosan to form a chitosan-acetate salt complex film. In both cases, the bands assigned to chitosan in the complex and the spectrum as a whole shift to lower wavenumbers, indicating CHI–salt interactions [49]. Then, (4) the amide II group band shifts to as low as 1540 cm^−1^, also related to N–H bending vibration and the NH_3_^+^ symmetric deformation. Moreover, (5) bands in the range from 1404 to 1307 cm^−1^ were attributed to the methylene and methyl groups, and (6) between 1151 and 1021 cm^−1^ were related to the C–O group stretching from glycosidic bonds. Finally, the peaks between 1250 and 800 cm^−1^ correspond to the fingerprint of polysaccharides like chitosan. For comparison, the spectrum of the sodium caseinate is also displayed in Figure 2. Also, for caseinate, signals at 3500–3200 cm^−1^ and at 2933 cm^−1^ are observed, due to the O–H and C–H stretching vibrations, respectively. Then, the main absorption peaks of sodium caseinate are identified between 1390 and 1700 cm^−1^. Within these bands, the stronger peak positions can be divided into amide I (1700–1600 cm^−1^) and amide II (1560–1500 cm^−1^). Specially, the amide I (1700–1600 cm^−1^) is broad as it is sensitive to the structure of the protein backbone mainly due to the transition dipole coupling, which after deconvolution could be used to analyze the compositional secondary structure of proteins. Some of the characteristic absorptions of chitosan and caseinate overlap to each other. Specifically, the FTIR spectra of chitosan–acetate and chitosan–caseinate casted materials show similar absorption patterns. In summary, the peaks in the region 1630–1540 cm^−1^ are more defined when going from net chitosan-acetate films to those consisting of chitosan and caseinate, as this band region is normally assigned to the amide group, which suggests the interaction of amino groups of chitosan with carboxyl groups as expected in films of chitosan-acetate and of chitosan–caseinate. Dhanasingh et al. [47] also showed that, as the ratio of chitosan/caseinate decreased, the dominant peaks of chitosan were observed to decrease, confirming the incorporation of caseinate during chitosan/caseinate coacervation.

#### 2.2.2. Raman Spectroscopy

**Figure 3:**
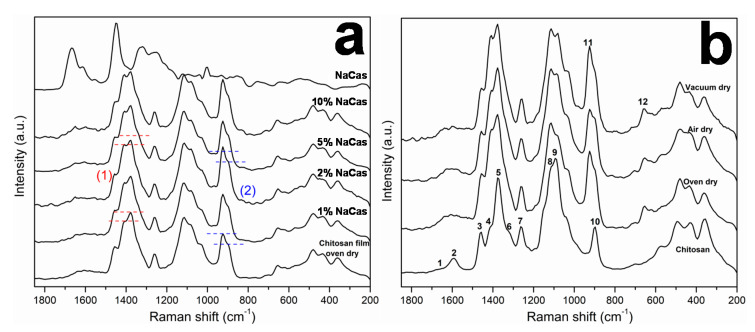
(**a**) Raman spectra of oven dry chitosan/caseinate films obtained after addition of different percentages of sodium caseinate (NaCas) to chitosan (HMW CHI), showing the increase of the intensity of the bands at 1410 and 1372 cm^−1^ ((1) in red), and the bands at 924 and 895 cm^−1^ ((2) in blue), when compared to the spectrum of the oven dry chitosan film without caseinate. The spectrum of the pure caseinate (NaCas) film is included for comparison. (**b**) Raman spectra of chitosan/acetate films obtained under different drying conditions. For comparison, the spectrum of the starting HMW CHI powder (chitosan) is also included in (**b**). The assignment of the bands regions 1, 2, 3, 4, 5, 6, 7, 8, 9, 10, 11, and 12 are discussed in detail above in the text in Section 2.2.2.

Figure 3a shows the Raman spectra of chitosan/caseinate casted films containing different caseinate concentrations, in comparison to those of films of chitosan and sodium caseinate. Figure 3b shows the Raman spectra of the chitosan powder (raw material) and of the films of chitosan/acetate formed from the casting of HMW CHI solubilized in aqueous acetic acid. The spectra of the chitosan/caseinate were similar to the chitosan/acetate film produced without the protein, since the amount of sodium caseinate is low and there are overlapping of the vibrational bands of caseinate and those ones of chitosan acetate. Differences in the relative intensity of the chitosan/caseinate vibrational bands could be observed when compared with the chitosan/acetate films, like the increase of the intensity of the bands at 1410 and 1372 cm^−1^ ( Figure 3a, (1)) and the bands at 924 and 895 cm^−1^ (Figure 3a, (2)). In the region 1630–1590 cm^−1^, a small increase of the intensity of the broadband (caseinate also has characteristic vibrational bands in this region) could be also observed [50]. The interactions between chitosan and caseinate can be masked by the presence of acetate from acetic acid and make difficult their visualization using spectroscopic techniques such as FTIR and Raman. Thus, the film formation route influences the appearance of chitosan spectra (acetate content, neutralization degree and crystallinity) [51]. In the case of chitosan powder, the following vibrational bands can be observed: (1, 2) at 1630 cm^−1^ a small shoulder corresponding to C=O stretching from amide (DA 3%) and a band at 1590 cm^−1^, corresponding to the NH_2_ in plane bending in chitosan; (3, 4, 5) band of CH_n_ groups, at 1457 cm^−1^ corresponding to the C-H asymmetrical vibration, a shoulder at 1407 cm^−1^ and an intense band at 1376 cm^−1^ corresponding to C-H, CH_2_, CH_3_ symmetric deformation, and ring vibration; (6) a shoulder at 1320 cm^−1^ corresponding to CN stretching and C-H in plane vibration; (7) at 1256 cm^−1^, C-C stretching and C-H in plane vibration; the bands (8) at 1113 cm^−1^ and (9) at 1087 cm^−1^ are related to the glycoside ring vibration; (10) a single band appears at 896 cm^−1^ corresponding to ring vibration and CH_2_ rocking [52]. The spectrum of chitosan acetate film is distinguished by differences in the intensities of the chitosan characteristic bands due to the presence of acetic acid and consequently the protonation of the amino groups of chitosan, as for example, the decreasing of the band at 1590 cm^−1^ (NH_2_) and increasing of the band at 1630 cm^−1^ due to the protonation of amino groups by acetic acid and the presence of C=O group also from acetic acid [50]. Another example is the shoulder at 1407 cm^−1^ which intensity increases for chitosan film due the vibration of the carboxylate ion (-COO^−^) [50]. A new band at 924 cm^−1^ (11) appears in the spectrum of chitosan acetate film and corresponds to the methyl group vibration from the acetic acid molecule, and the band at 896 cm^−1^ in the chitosan powder shifts to 903 cm^−1^ in the chitosan acetate film [52]. Finally, a new band is observed at 654 cm^−1^ (12) corresponding to the OC=O band (in-plane vibration mode) of acetate.

### 2.3. Characterization of Chitosan-Caseinate Polyelectrolyte Complex Systems

Case I: Complexation by Addition of NaCas into Chitosan in Excess

**Figure 4:**
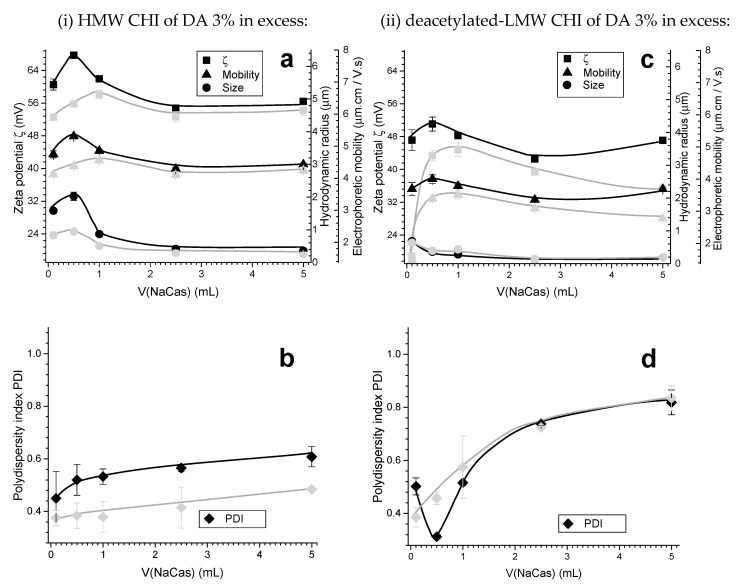
Zeta potential ζ, electrophoretic mobility, hydrodynamic radius (size) and its polydispersity index PDI respectively obtained for the experiments of the Case I (chitosan in excess): (i) sodium caseinate added to HMW CHI of low DA = 3% (**a**,**b**); (ii) sodium caseinate added to deacetylated-LMW CHI of low DA = 3% (**c**,**d**). Black curves show the polyelectrolyte complex PEC characteristics obtained in the first week; grey curves show the results obtained after two weeks aging at room temperature.

**Figure 5:**
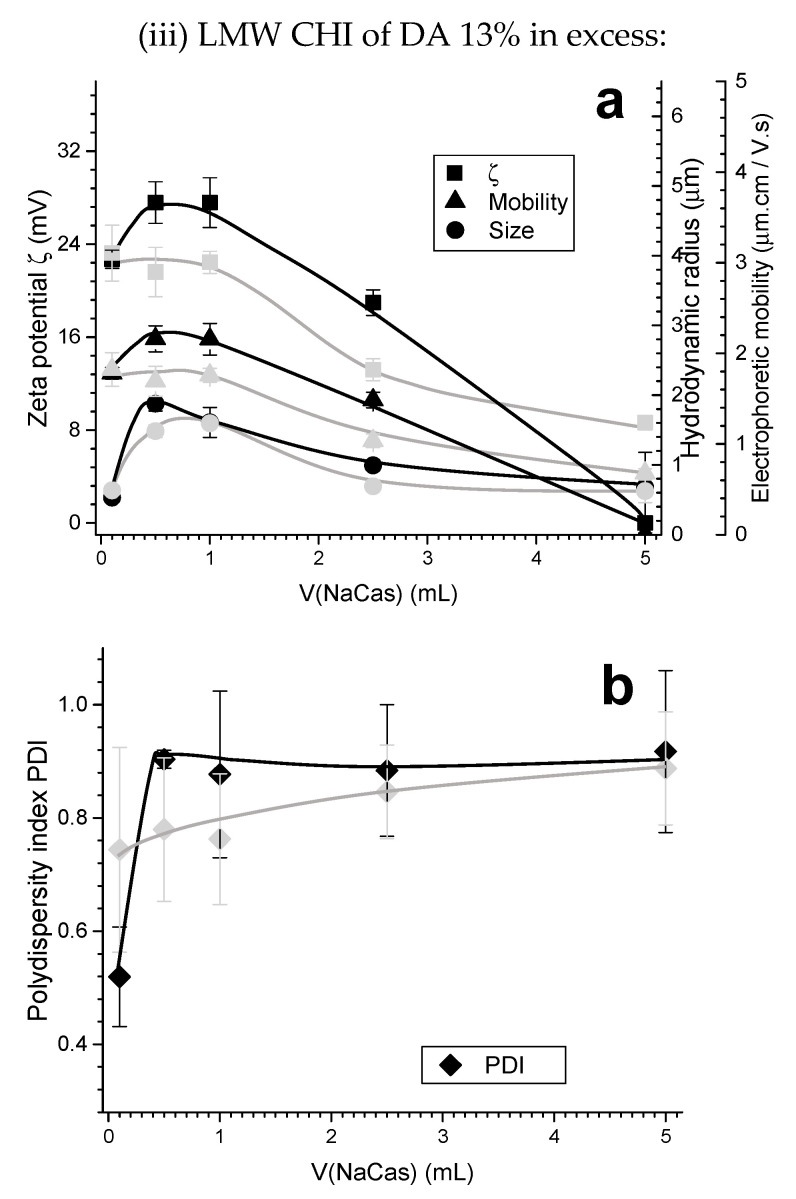
(**a**) Zeta potential ζ, electrophoretic mobility, hydrodynamic radius (size), and (**b**) polydispersity index PDI obtained for the experiment (iii) of the Case I (chitosan in excess): sodium caseinate added to LMW CHI of high DA = 13%. Black curves show the polyelectrolyte complex (PEC) characteristics obtained in the first week; grey curves show the results obtained after two weeks aging at room temperature.

Figure 4 and Figure 5 show the zeta potential, hydrodynamic radius, size polydispersity index (PDI) and electrophoretic mobility of the colloidal systems obtained in the three experiments: (i) with HMW CHI of low DA, (ii) with deacetylated-LMW CHI of low DA, and (iii) with LMW CHI of high DA, in which given volumes of 0.1% (w/w) sodium caseinate were added to a fixed volume of 0.1% (w/w) chitosan solution initially in excess, as described in Section 4.5 in Materials and Methods and Table 2. With the addition of caseinate to the excess of chitosan, the mixture appeared homogeneous to naked eye. Moreover, after its centrifugation at 2000 rpm for 20 min, in general no sediment was observed in the different experiments. Then, 3.50 mL of the ‘supernatant’ were collected and the surface characteristics of the CHI–caseinate polyelectrolyte complex (PEC) system were investigated using a Zetasizer.

#### 2.3.1. Effect of Chitosan Molecular Weight

The addition of NaCas to high molecular weight chitosan (HMW CHI) yields a stable colloidal system. It was observed that the zeta potential and hydrodynamic radius both achieved a maximum value (+68 mV and 2 µm, respectively), when the volumetric ratio of NaCas:HMW CHI was 1:10, after which it decreased as the added amount of NaCas increased (Figure 4a). The initial aqueous solution of 0.1% (w/w) of HMW CHI of low DA = 3% exhibited a zeta potential of around +60 mV. Then, the zeta potential always remained positive, even after the addition of higher caseinate amounts into chitosan solution. Similar trends for the electrophoretic mobility were observed, with the highest mobility obtained after adding 0.10–0.50 mL of 0.1% (w/w) aqueous NaCas. The measured size polydispersity index (PDI) of the PEC system was 0.3–0.6 (± 0.1) and it increased with the caseinate amount (Figure 4b).

However, when deacetylated low molecular weight chitosan (deacetylated-LMW CHI) also with low DA = 3% was used in the experiment (ii), lower hydrodynamic radii were obtained (Figure 4c). After adding 2.50 mL of 0.1% (w/w) caseinate, the hydrodynamic radius reached a plateau at around 200 nm. The zeta potential values stayed positive, but were lower (between +47 and +52 mV, Figure 4c) than the values obtained above with the HMW CHI (Figure 4a). The sharp maximum at low NaCas amount was not observed for the zeta potential and this value tended to decrease with aging of the PEC system (Figure 4c, grey). A similar trend was observed for the electrophoretic mobility. The size PDI significantly increased upon the addition of NaCas which was concomitant with a decrease of the PEC hydrodynamic radius (Figure 4d).

#### 2.3.2. Effect of Chitosan Degree of Acetylation (DA)

A comparison of Figure 4c,d and Figure 5 evidences the impact of varying the chitosan degree of acetylation (DA) from 3 to 13% with CHIs having similar molecular weight. It was observed that after adding 1.00 mL of 0.1% (w/w) NaCas to 0.1% (w/w) LMW CHI of higher DA = 13% in the experiment (iii), the zeta potential starts to decrease, and so does the electrophoretic mobility (Figure 5a). At higher amounts of added NaCas, the colloidal system becomes unstable and tends to coagulate, as for a volumetric or mass ratio of NaCas:CHI of 1:1 (i.e., at V(NaCas) = 5.00 mL) the zeta potential achieved values around zero (Figure 5a). A very different behavior was observed with a DA of 3% (Figure 4c). Thus, the degree of acetylation (DA) plays a significant role in the stability of particles, possibly determining the structure of the PEC formed. In this system, at a DA of 13%, the hydrodynamic radii were higher and the PDI was also high (Figure 5), which suggests particle aggregation.

Case II: Complexation with Sodium Caseinate in Excess

**Figure 6:**
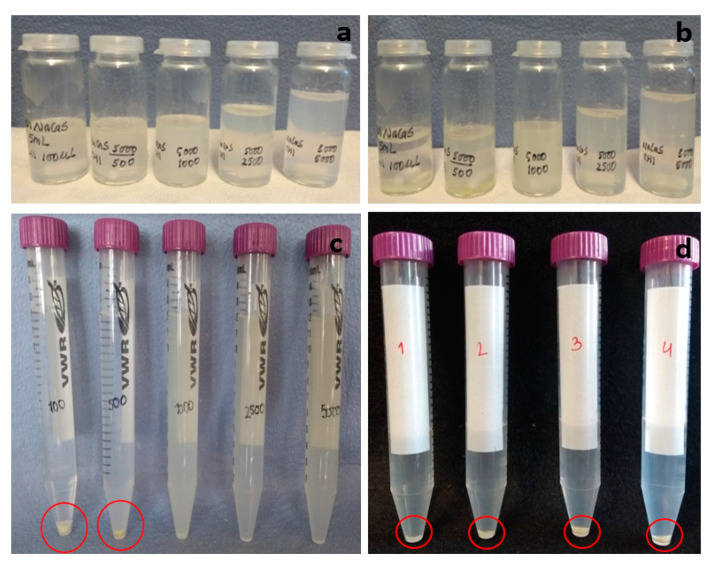
(**a**) Polyelectrolyte complex (PEC) colloidal systems obtained in the experiment (iv) of Case II (caseinate in excess) after adding 0.10, 0.50, 1.00, 2.50, or 5.00 mL of 0.1% (w/w) high molecular weight chitosan HMW CHI solution into 5.00 mL of 0.1% (w/w) of sodium caseinate. (**b**) PEC colloidal systems obtained after furtherly adding 5 µL of the crosslinker glutaraldehyde (GLU). (**c**) Photos showing the sedimented material collected after centrifugation of the PEC colloids shown in (**a**); where sedimented centrifugate (highlighted in red circle) could be collected when lower volumes of 0.10 and 0.50 mL of 0.1% (w/w) chitosan were added. (**d**) Photos showing the sedimented material (highlighted in red circle) collected after centrifugation of the PEC colloids crosslinked with GLU and obtained with intermediate volumes of 0.10, 0.20, 0.50, and 0.75 mL of 0.1% (w/w) HMW CHI added to 0.1% (w/w) NaCas.

In these studies in which a given volume of 0.1% (w/w) chitosan was added to a volume in excess of 0.1% NaCas sub-micellar dispersion system, as described in Section 4, for the experiments of Case II: (iv) with HMW CHI of low DA, (v) with deacetylated-LMW CHI of low DA, and (vi) with LMW CHI of high DA, it was observed that the system turned milky when 0.10 to 5.00 mL of 0.1% (v/v) chitosan solution were added to the caseinate dispersion system (Figure 6). Upon centrifugation at 2000 rpm for 20 min, a distinct sedimented material was obtained for the experiments where the volumetric ratio of CHI:NaCas were 1:50 and 1:10, as displayed in the photographs of Figure 6c. As the added CHI volume was further increased, the amount of collected centrifugate was negligible and a turbid supernatant remained, revealing a stable colloidal system consisting of small nanoparticles. To verify the formation of distinct nanoparticles, also in these latter conditions, the crosslinker glutaraldehyde (GLU) was added to the chitosan–caseinate mixture to chemically crosslink both the chitosan and caseinate, and thereby further promote the hardening of the NPs. Figure 6b shows the achievement of colloidal systems also after adding GLU. Then, Figure 6d shows that a higher amount of sedimented material could be collected after centrifugation when adding the GLU crosslinker.

#### 2.3.3. Effect of Chitosan Molecular Weight

**Figure 7:**
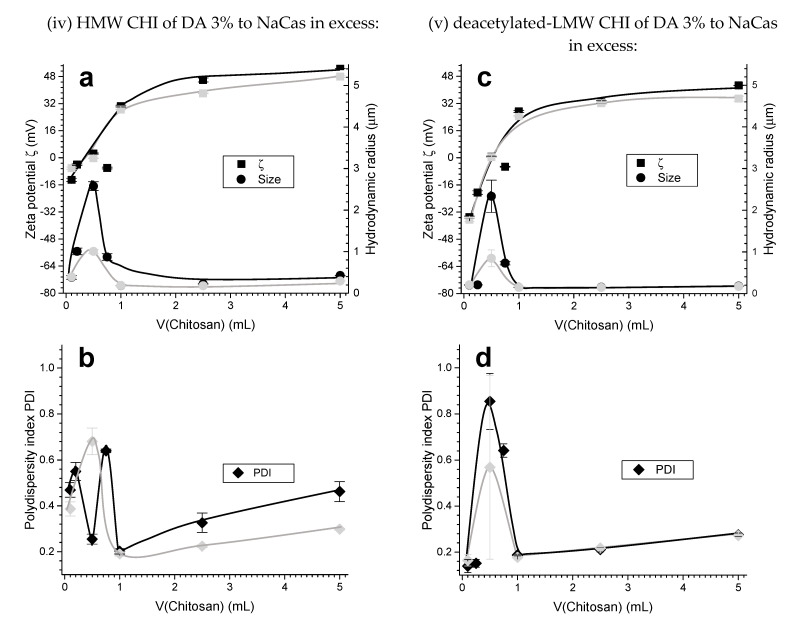
Zeta potential ζ, hydrodynamic radius (size) and its polydispersity index PDI respectively obtained for the experiments of the Case II (caseinate in excess): (iv) HMW CHI of low DA = 3% added to NaCas (**a**,**b**); (v) deacetylated-LMW CHI of low DA = 3% added to NaCas (**c**,**d**). Black curves show the characteristics of the chitosan-caseinate PEC colloidal system obtained in the first week; grey curves show the results obtained after two weeks aging at room temperature.

When chitosan solution was added to a sodium caseinate dispersion system in excess, the zeta potential changed relatively fast from a negative to positive value (Figure 7). A sharp increase in hydrodynamic radius (approximately 2.5 μm) was observed upon the addition of 0.50 mL of HMW CHI, when the zeta potential was very low and close to zero. At this point, electrostatic stabilization of the colloids is no longer efficient, and the particles become unstable and form aggregates. Thereafter, the value of zeta potential and consequently the electrophoretic mobility steadily increased, up to the zeta potential of the corresponding mirror (Case I) systems. Figure 7a shows the plot of zeta potential and hydrodynamic radius as a function of added volume of 0.1% (w/w) HMW CHI of low DA = 3% to 0.1% (w/w) NaCas. The hydrodynamic radius of the PEC colloidal particles dropped to around 200 nm after addition of 1.00 mL of HMW CHI and practically remained at this value as the added volume of chitosan further increased. A similar trend was observed when low molecular weight chitosan also of low DA = 3% (deacetylated-LMW CHI) was used (Figure 7c).

**Figure 8:**
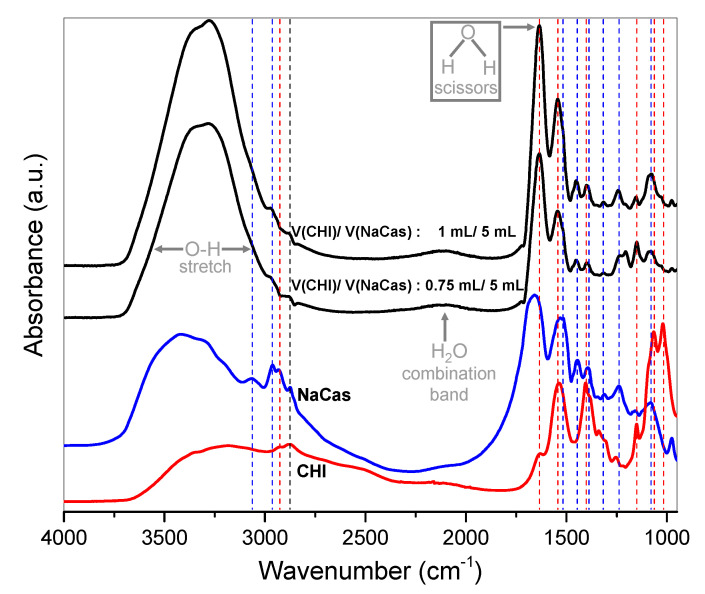
ATR-FTIR spectra of chitosan-caseinate hydrogel nanoparticles (NPs) collected in the centrifugates of the experiment (iv) of Case II (caseinate in excess), in which 0.75 or 1.00 mL of 0.1% (w/v) solution of HMW CHI of low DA = 3% were respectively added to 5.00 mL of 0.1% (w/v) NaCas sub-micellar dispersion system. The ATR-FTIR spectra of the chitosan acetate (red) and sodium caseinate (blue) are also displayed, and the dashed lines (red or blue, respectively) highlight the relevant peaks of these polymer constituents in the CHI-caseinate hydrogel NPs. The grey arrows highlight the absorption bands of the water present in the hydrogel NPs.

The attenuated total reflection–Fourier transform infrared (ATR-FTIR) analyses of Figure 8 show the characterization of the chemical structure of the hydrogel nanoparticles (NPs) collected after centrifugation, which is exemplarized for the NPs obtained in the experiment (iv) of Case II conditions. The ATR-FTIR spectra demonstrate that the collected nanoparticles contain chitosan, caseinate, and water, as expected for chitosan–caseinate hydrogel nanoparticles, also with the peak positions of the caseinate well observed. When increasing the added volume of chitosan the absorption peaks of chitosan become stronger. Further, here, for the chitosan–caseinate nanoparticles, as for the above discussed chitosan–caseinate films, the carbonyl band usually observed at ~1670 cm^−1^ for chitosan in its free amine form shifts to lower wavenumbers around 1630 cm^−1^ associated to the amide I group and NH_3_^+^ asymmetric deformation, when chitosan interacts with caseinate to form CHI–caseinate complex nanoparticles. As above, the bands assigned to CHI in the complex and the spectrum as a whole shift to lower wavenumbers, indicating CHI–salt interactions.

**Figure 9:**
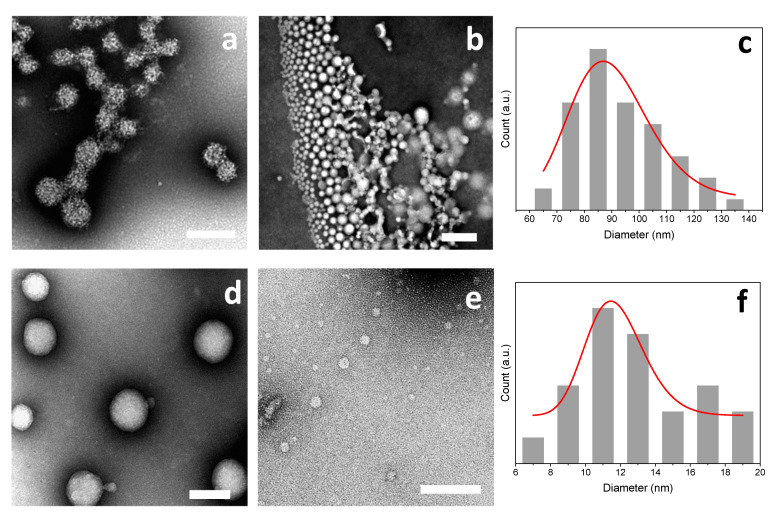
TEM micrographs of chitosan-caseinate PEC nanoparticles obtained in the experiments of Case II (caseinate in excess): (iv) by adding 0.1% (w/w) HMW CHI of low DA = 3% to 0.1% (w/w) sodium caseinate in CHI:NaCas volume ratios 1:10 (**a**) and 1:2 (**b**), with the corresponding dried nanoparticle size distribution (**c**) for CHI:NaCas volume ratio 1:2; (v) by adding 0.1% (w/w) deacetylated-LMW CHI of low DA = 3% to 0.1% (w/w) sodium caseinate in CHI:NaCas volume ratios 1:10 (**d**) and 1:2 (**e**), with the corresponding nanoparticle size distribution (**f**) for CHI:NaCas volume ratio 1:2. Scale bars: (a; b) 500 nm; (**d**) 200 nm; (**e**) 100 nm.

The morphology of the PEC nanoparticles (NPs) collected in the centrifugates obtained in Case II conditions (caseinate in excess) were examined using transmission electron microscopy (TEM). For all experiments, the dried particles were observed to be spherical, and the NPs size distribution could be obtained from image analysis of the TEM micrographs. Figure 9a,b shows chitosan–caseinate NPs obtained in the experiment (iv) with high molecular weight chitosan (HMW CHI) of low DA added to NaCas in excess. Figure 9d,e illustrates TEM micrographs of nanoparticles obtained in the experiment (v) where a low molecular weight chitosan of low DA (deacetylated-LMW CHI) was added to NaCas in excess.

The TEM characterization of the nanoparticles morphology reveals that they were well individualized (non aggregated) and highly spherical in shape (Figure 9). The size of particles changed as the ratio of CHI:NaCas varied. In general, it was observed that on increasing the added amount of CHI the particle size decreased, as evidenced by hydrodynamic gyration radius evaluations. The analysis of the TEM images revealed, for example, porous chitosan–caseinate particles with the biggest size average of 200 nm (Figure 9a) for the nanoparticles obtained in the experiment (iv) of Case II by adding 0.50 mL of 0.1% (w/w) high molecular weight chitosan HMW CHI of low DA = 3% to 0.1% (w/w) sodium caseinate (CHI:NaCas volume ratio of 1:10). When a higher CHI:NaCas volumetric ratio of 1:2 was used, smaller dried particles were obtained with an average diameter of about 95 nm (Figure 9b,c).

In the experiment (v) of Case II in which low molecular weight chitosan (deacetylated-LMW CHI) also of low DA = 3% was added to NaCas (CHI:NaCas~1:10), it was also observed that the nanoparticle size decreased as the added amount of chitosan increased. In this experiment, particle average diameters close to 80 nm (Figure 9d), comparatively smaller than those obtained above with high molecular weight chitosan were obtained. Then, adding chitosan to achieve a CHI:NaCas volume ratio of 1:2 led to smaller particles with mean diameter ~15 nm (Figure 9e,f).

#### 2.3.4. Effect of Chitosan Degree of Acetylation (DA)

**Figure 10:**
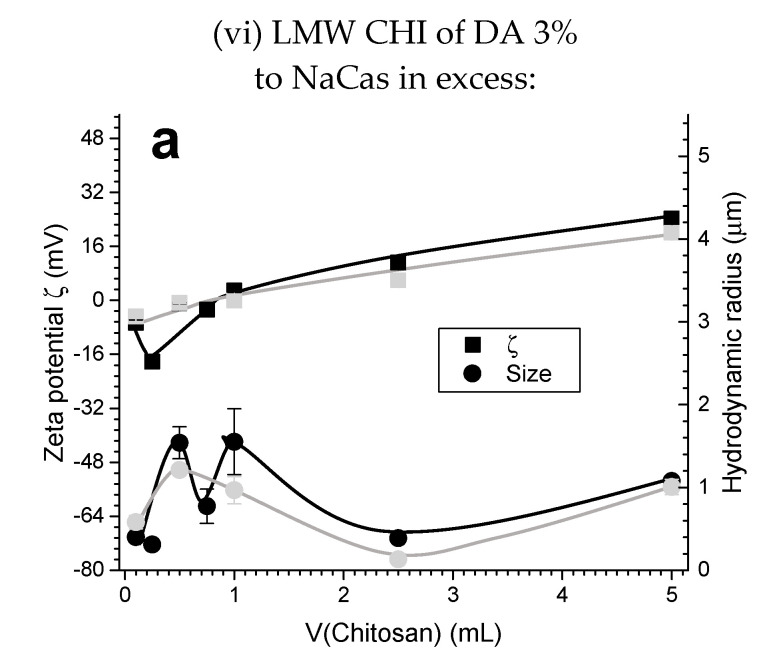

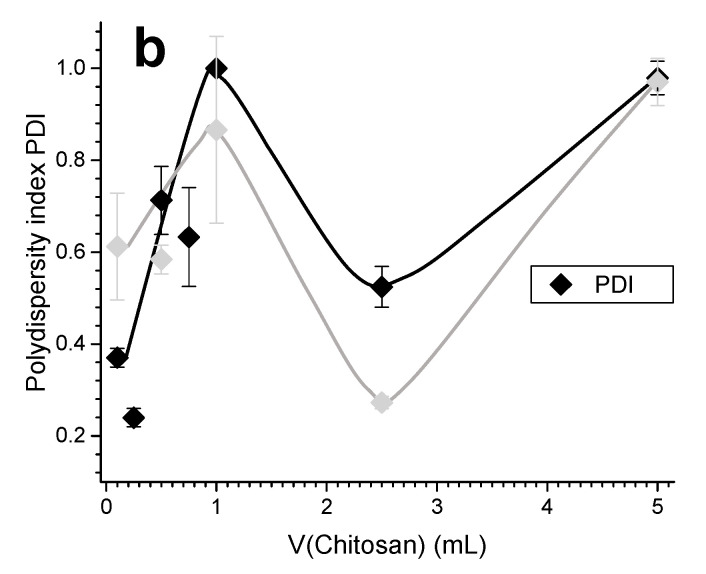
(**a**) Zeta potential ζ, hydrodynamic radius (size), and (**b**) polydispersity index PDI obtained for the experiment (vi) of the Case II (caseinate in excess): LMW CHI of high DA = 13% added to caseinate. Black curves show the characteristics of the chitosan-caseinate PEC colloidal system obtained in the first week; grey curves show the results obtained after two weeks aging at room temperature.

Figure 7c,d and Figure 10 show the impact of chitosan DA (3% and 13%) for similar molecular weight chitosans. It is observed that the zeta potential decreases as the DA increases.

**Figure 11:**
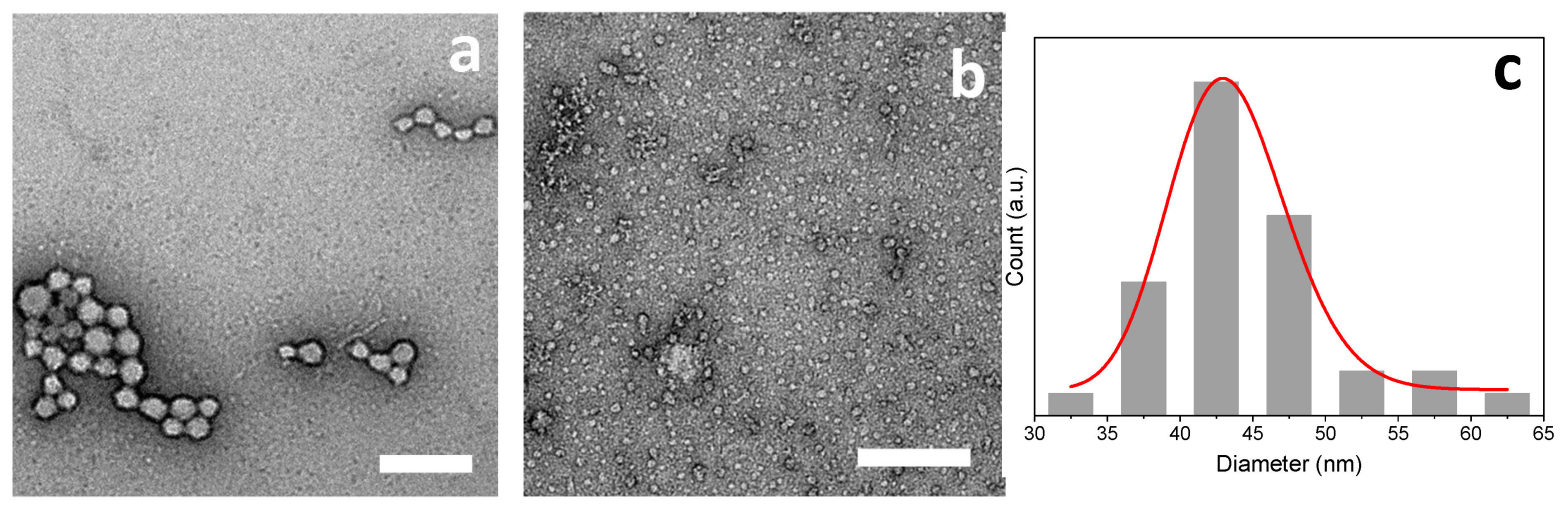
TEM micrographs of of chitosan-caseinate PEC nanoparticles obtained in the experiment (vi) of Case II (caseinate in excess) by adding 0.1% (w/w) solution of LMW CHI with DA = 13% to 0.1% (w/w) sodium caseinate in CHI:NaCas volume ratios of 1:10 (**a**) and 1:2 (**b**), with the corresponding dried nanoparticles size distribution (**c**) for CHI:NaCas volume ratio 1:2. Scale bars: (a; b) 200 nm.

TEM images of the chitosan–caseinate nanoparticles obtained in the experiment (vi) of Case II (LMW CHI of high DA = 13%) are displayed in the Figure 11. Nanoparticles size averages of 45 and 20 nm were obtained when the volume ratio of LMW CHI to NaCas solution was 1:10 and 1:2, respectively (Figure 11). The achievement of CHI–caseinate PEC nanoparticles seems to be relatively robust, independently of the structural parameters of chitosan. Dry particle rehydration and redispersion would deserve to be further evaluated, as well as the stability of CHI–caseinate nanoparticles in physiological media.

### 2.4. Insulin Delivery Chitosan-Caseinate Nanoparticle Systems. Insulin Entrapment Efficiency and Release

**Scheme 1:**
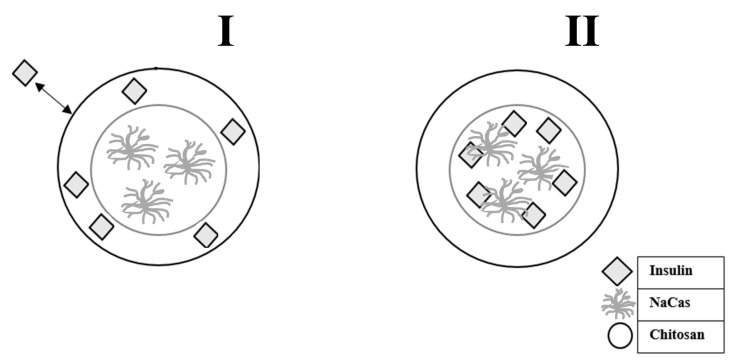
Two models hypothesized for insulin entrapment in chitosan-caseinate PEC nanoparticles, both of them corresponding to the experiment (vi) (high molecular weight chitosan of low DA, HMW CHI) of the Case II (caseinate in excess). Model **I**: insulin is initially mixed with the chitosan, which mixture is then added to the caseinate in excess. Model **II**: insulin is initially mixed with the caseinate in excess, onto which mixture the chitosan is then added.

To prepare insulin entrapped chitosan–caseinate nanoparticles, two approaches were considered. In Model I, the insulin was hypothesized to first bind to the chitosan to constitute a loaded particle shell, with the caseinate located in the nanoparticle core region (Scheme 1-I). In Model II the insulin was firstly put in interaction with the sodium caseinate solution, which mixture was then expected to be surrounded by a chitosan shell (Scheme 1-II). In both approaches, the chitosan of high molecular weight and low DA (HMW CHI) was used, and experiments corresponding to Case II (chitosan solution added to NaCas in excess) were considered.

The free insulin, which was delivered into the supernatant after centrifugation of the insulin loaded chitosan–caseinate nanoparticles (NPs), was determined. Similar insulin entrapment efficiencies could be determined in NPs processed following the routes of Model I and II (Scheme 1). By using Equation (2) (Section 4), the estimated insulin entrapment efficiencies in the NPs were 77.4% and 75.6% for Models I and II, respectively.

**Figure 12:**
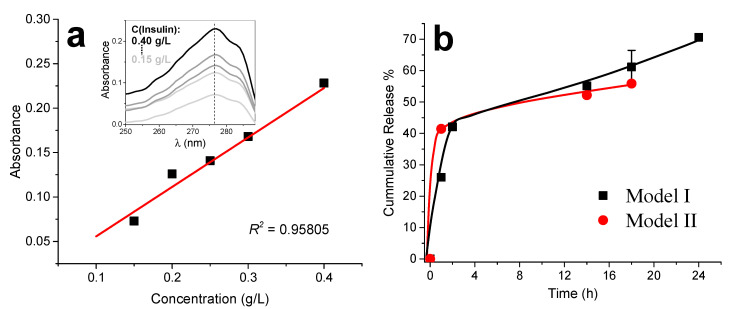
(**a**) Insulin standard calibration curve obtained by UV spectrophotometry for the absorbance measured at 276 nm wavelength. Inset shows the UV spectra obtained for the different insulin standard concentrations. (**b**) Cumulative insulin release from chitosan-caseinate nanoparticles obtained following the Model I and II approaches of Scheme I, as determined by UV spectrometry at 276 nm wavelength**.**

The calibration curve obtained by UV spectroscopy for the used insulin concentrations is shown in Figure 12a. In the insulin release studies in phosphate-buffered saline (PBS) at physiological pH 7.4, we observed a boost-release effect in the first two hours of release. The cumulative drug release for Model I in the first hour was around 26.0% while that for Model II was around 41.5%. This is an indication that the loading topology of the nanoparticles could have a minor impact. The kinetics of insulin release in the earlier times in Model I would be determined by the pre-establishment of molecular interactions between (protonated) chitosan and insulin before polyelectrolyte association. For longer release times, the cumulative release values of Model I overcome those of Model II and continue their gradual increase, with release as high as 70.6% observed at 24 h for Model I. If chitosan-insulin interactions are governing the release kinetics, it is expected that the protonation state of chitosan should play its role, both on the structure of the corona and the strength of chitosan–insulin interaction. We envision that the physicochemical properties of the release medium (pH and ionic force) will have a major impact. In particular, further studies in the conditions simulating the gastrointestinal tract are ongoing.

## 3. Discussion

### 3.1. Effect of Chitosan Molecular Weight, Degree of Acetylation (DA) and Order of Addition of Polyelectrolytes on the Chitosan-Caseinate PEC Colloidal System Characteristics

In Case I, in which the sodium caseinate (NaCas) was added to the chitosan in excess, as mentioned above, nanoparticles could not be collected in the relatively mild conditions used for centrifugation. In Case I, both the CHI molecular weight and the degree of acetylation (DA) play an important role in the formation of stable colloidal systems. For chitosan of high molecular weight (HMW CHI) with almost no acetylation (low DA = 3%), the achieved colloid presents a relatively high particle surface charge, resulting in positive zeta potential values with even a maximum value for disordered PECs. Whatever used chitosan, the formation of the particles starts even at low caseinate amounts added to the CHI in excess. More stable and smaller particles were progressively obtained still with positive zeta potential by increasing the added amount of caseinate, which shows that the NPs surface is constituted by the polycation, i.e., CHI chains forming a nanocoating, since the polyelectrolyte character of highly deacetylated CHI (rather strong polycation) is dominating the zeta potential over that of NaCas. This leads to the formation of particles with a distinct surface bearing a positive charge due to the NH_3_^+^ groups of chitosan. Since the apparent pKa of almost fully deacetylated chitosan is in the range 6.2–6.5, the apparent charge may be impacted by pH, in particular in physiological fluids in presence of salts. Finally, in the physicochemical environment chosen for the PEC association at acidic pH, very stable colloids are formed.

According to the results of the experiments (i–iii) of the Case I, two NP formation regimes can be identified at adding a dispersed NaCas sub-micellar system into CHI solution. At low NaCas added volumes, large particles form with high zeta potential. These primary PECs are far from equilibrium and may result from caseinate aggregates externally neutralized and bridged with CHI chains. Aging promotes the fragmentation, dispersion, and stabilization of NaCas sub-micelles with chitosan corona electrostatically strongly interacting with the anionic caseinate core. Larger amounts of added NaCas also yields smaller secondary PECs nanoparticles as previously described for other PECs obtained with synthetic polymers [53]. Chitosan chains act to stabilize the nanoparticles sterically and electrostatically. In the caseinate core/CHI corona model, smaller chains could induce the formation of a thinner corona as a result of a stronger adsorption of shorter chains. Increasing the DA from 3% to 13% for low molecular weight chitosan strongly impacts the size of the PECs nanoparticles. This could result from: (i) a decrease of CHI charge density which is less favorable to PEC association and nanoparticle stabilization, (ii) a decrease of caseinate micellar aggregate fragmentations and (iii) more extended chain conformations in the CHI corona.

When the low molecular weight CHI of low DA (deacetylated-LMW CHI) is used at NaCas:CHI weight mixing ratios close to 1:2, lower hydrodynamic radii are obtained with a plateau achieved at around 200 nm. These robust and optimal conditions are reached thanks to the relatively high charge density of chitosan and the pre-arrangement of the sodium caseinate as sub-micelles. A good stability is observed even if the zeta potential values stayed positive but remained lower than those obtained with the high molecular weight CHI of low DA. For low molecular weight CHI, the formation of primary disordered PECS (sharp maximum of zeta potential vs. added volume of NaCas) was not observed, and the aging in aqueous solution decreases the size of the colloidal particles into a more stable PEC association with decrease of the surface charge (zeta potential decreases after two weeks, which effect is more significant when adding more caseinate into the chitosan).

In relation to the CHI degree of acetylation (DA), in contrast to the results observed with low acetylated CHI (strong polycation) (Figure 4), a relatively high DA (i.e., 13%) easily allows to decrease the initial positive zeta potential of CHI under addition of caseinate. Apparently, a strong charge density of chitosan is necessary to avoid the formation of unstable colloids with a tendency to coagulate. Specially, when the weight of added caseinate is equal to the CHI weight, complete phase separation is observed, with apparent zeta potential equal to zero (Figure 5). Thus, the CHI degree of acetylation plays a major role in the nanoparticle stability, possibly determining the establishment of strong PEC associations. In general, higher hydrodynamic radii and polydispersity indexes are obtained when using chitosan of high DA, suggesting particles aggregation and poor colloidal stability.

In Case II, in which the chitosan is added to the caseinate initially in excess, the turbidity of the developing colloidal system is clearly visible. Representative amount of sedimented nanoparticles is observed for low quantities of added CHI, which can be easily collected even with a relatively mild centrifugation at 2000 rpm for 20 min. In fact, the initial negative charge of CHI–caseinate nanoparticles could be easily reversed by adding low CHI amounts into caseinate in excess, the effect of which was more significant for the chitosans of low DA, which again showed that the strength of the cationic and anionic paired polyelectrolytes do not play the same role in the association. Upon adding higher amounts of high molecular weight chitosan of low DA, the colloid becomes more stable and it is more difficult to distinguish any initial sedimented material. Thus, primary to secondary PECs structural transition also occurs in these processing conditions. In fact, in these Case II conditions, the zeta potential increases from a negative to a positive value, inducing a sharp increase in hydrodynamic radius and polydispersity index (Figure 7), indicative of a lower colloidal stability. This is evidence that the stability of the colloid is due to electrostatic forces, thanks to the addition of the cationic chitosan polyelectrolyte, contributing to an outer positive shell. In fact, the ATR-FTIR spectra of the obtained chitosan-acetate hydrogel nanoparticles revealed representative amount of caseinate in the NPs and that the chitosan contained in the NPs could be increased by adding more chitosan into the caseinate solution (Figure 8). Again, this phenomenon seems to be related to both the sub-micelles already preformed in the starting NaCas dispersion system and the strong polyelectrolyte character of CHI; which reflects the need of just adding small aliquots of CHI to cover the preformed caseinate sub-micelles to yield CHI–caseinate PEC nanoparticles. For instance, very small PEC colloidal nanoparticles of hydrodynamic radius of around 200 nm can be obtained by just adding 20% weight of chitosan respect to caseinate weight. Again, the size of nanoparticles remains small even under further addition of CHI when the DA is low and for both high and low CHI molecular weights (Figure 7). A similar trend was observed when further analyzing the nanoparticles in the dried state by TEM (Figure 8).

Other PEC systems from polymer solutions formed in different conditions might exhibit an opposite trend with the formation of primary complexes aggregating into larger secondary polyelectrolyte associations [54]. In several well documented works [55,56], an efficient PEC formation strategy has been reported when a high molar mass polymer is used in excess, acting as a host while the other shorter polyelectrolyte acts as a guest. The formation of PEC systems from soluble polymers are thus governed by guest-host interactions in which the formation of the particles proceeds via charge neutralization and collapse of the assembled poylelectrolyte at the nanoscale. In such a scenario, the excess polymer may form the outer shell while the core is formed by the neutral complex segments. In our case however, this model could not capture all the observed features of CHI chains interactions with caseinate sub-micelles [40,57]. Since the caseinate sub-micelles should be preformed themselves in their original dispersion system [40], CHI should cover the aggregate of a few elementary caseinate micellar nanodomains. Thus, the molecular weight, flexibility, and charge density of CHI in solution have an important role on the achieved polyelectrolyte association structure. In the Case II conditions, as expected the zeta potential also decreases as the DA increases (Figure 10, DA = 13%). These results reveal that a relatively slight increase in the DA (from 3% to 13%), with the presence of few neutral N-acetyl glucosamine groups in CHI chains, leads to a decrease in the polyelectrolyte association strength, which in turn contributes to the formation of a loose corona constituted by CHI chains with a more expanded conformation. CHI ‘loops’ possibly could form when the electrostatic repulsions between NH_3_^+^ groups of the D-glucosamine units (CHI deacetylated units) are missing, the effect of which is highlighted in high DA chitosan.

Finally, the obtained chitosan–caseinate particles were observed to have relatively small sizes (about 200 nm) as desired for polymer nanoparticle applications in drug or gene delivery. However, some experiments suggest that agglomeration of the small nanoparticles can occur and some of them exhibit highly porous structure like derived from native caseinate sub-micellar structures already characterized by high-resolution field-emission scanning electron microscopy [57,58], and as suggested in this work after transmission electron microscopy observations (e.g., Figure 9).

### 3.2. Insulin Delivery from Chitosan-Caseinate Nanoparticles

Insulin is an amphoteric protein molecule containing many ionizable groups, distributed in six amino acid residues able to electrostatically attach to a positively charged polyelectrolyte like chitosan [28,44,59], and in ten amino acid residues able to attain a negatively charged polyelectrolyte like caseinate [44]. These properties could be responsible for the entrapment of insulin into the chitosan–caseinate nanoparticles. The obtained insulin entrapment efficiencies of 77.4% and 75.6% in the chitosan–caseinate nanoparticles obtained in Models I and II (see Scheme 1), respectively, suggest that the major interactions of insulin are established with the polycation chitosan [28,59,60]. The electrostatic interaction with the large amount of positively charged amino groups of the used HMW CHI should be favored, as this is a chitosan with almost no acetylation (DA = 3%) and higher charge density. The influence of chitosan DA on the insulin loading capacity of nanoparticles was also reported in studies where chitosan/TPP nanoparticles of comparative lower DA like 13% showed higher positive zeta potential values and higher insulin loading efficiency than nanoparticles prepared with chitosan of higher DA like 30% [60]. Chitosan of lower DAs (0–5%) as investigated in this study (e.g., DA = 3%) should exhibit a more accentuated behavior supporting stronger interaction between insulin and the positively charged polycation chitosan.

**Scheme 2:**
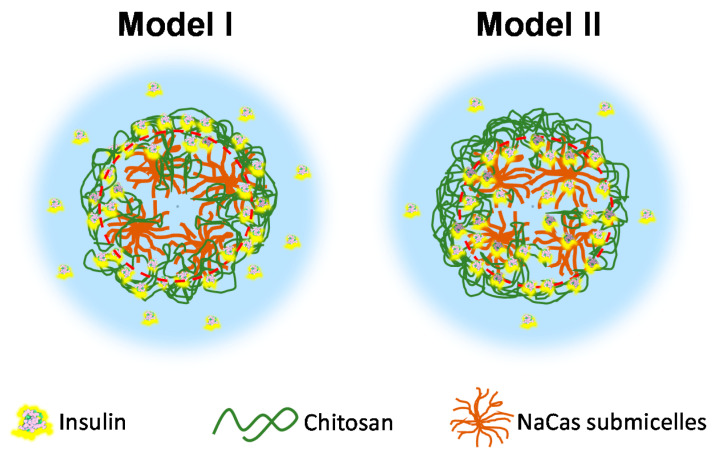
Schematic illustration of the release of insulin from the chitosan-caseinate nanoparticles in Models I and II. Left: Model I: insulin evenly distributed within the chitosan (CHI) shell surrounding the inner submicellar caseinate core, where the pre-established attachment and high affinity of insulin with highly deacetylated CHI in their preformed mixture play a delay role against the earliest release of insulin, but at the latest stages a loosening of insulin-polyelectrolyte interactions occur with faster release of the insulin mainly located in the particle CHI shell. Right: Model II: insulin mainly located in the inner part of the chitosan shell at the boundary of the caseinate submicellar core with some insulin dispersed within the caseinate, showing a more equilibrated diffusion of the initially “loose” insulin dispersed in the caseinate core, which should overcome the chitosan shell barrier with high affinity to insulin, before achieving release to the outer medium.

The cumulative release of insulin was higher in a first stage (at release times ~1 h) in Model II (where insulin firstly mixed with caseinate, on which mixture chitosan was added to possibly constitutes a shell), in comparison with association Model I (insulin firstly mixed with chitosan to possibly constitute the particle shell, which mixture was added on the caseinate constituting the nanoparticle inner region) (Figure 12). As represented in Scheme 2, the pre-established attachment and high affinity of insulin with highly deacetylated chitosan in their pre-formed mixture in Model I should play a delay role against the earliest release of insulin. After longer contact of the particles with the release medium (phosphate-buffered saline PBS, pH 7.4), in Model I, a loosening of insulin-polyelectrolyte interactions might occur with a faster release of the insulin mainly located in the particle CHI shell (Scheme 2, left), on one hand due to the competition between caseinate and insulin to bind chitosan, and on the other hand due to the pH of the medium being higher than the chitosan pKa (6.2–6.5), conditions which favor the neutralization of the positively charged NH_3_^+^ groups of chitosan. Thus, model II seems to be more appropriate to allow a controlled release of insulin from the chitosan–caseinate nanoparticles (Figure 12), with a more equilibrated diffusion of the initially “loose” insulin dispersed in the caseinate core, which at the longer times should overcome the chitosan shell barrier, with high affinity to insulin, before achieving release to the outer medium (Scheme 2, right). Finally, a relatively high drug release observed for both Models I and II could be due to the high porosity of the chitosan–caseinate nanoparticles, as observed by TEM and described by previous authors in systems containing micellar constituents [57,58] and to the release medium pH (7.4) considered in the study.

## 4. Materials and Methods

Sodium caseinate (NaCas) was kindly provided by Prof. Frédéric Prochazka (Lactips, Saint-Jean-Bonnefds, France) which was produced from bovine milk.

Chitosan of high molecular weight (HMW CHI) obtained from squid pen chitin and supplied by Mahtani Chitosan (type: Chitosan 144), Veraval, India, and of low molecular weight (LMW CHI) purchased from Sigma-Aldrich Taufkirchen, Germany were used as starting raw materials. Glutaraldehyde solution (50% w/w) was purchased from Sigma-Aldrich, Taufkirchen, Germany. Human insulin was purchased from Roche Diagnostics, Mannheim, Germany.

Chitosan deacetylation: The deacetylated low molecular weight chitosan, hereafter called deacetylated-LMW CHI, was prepared by using as raw material the above low molecular weight chitosan (LMW CHI) to achieve a degree of acetylation as low as that of the above high molecular weight chitosan (HMW CHI). The LMW CHI, initially having DA = 13%, was then processed to achieve almost full deacetylation by following the methodology of freezing, pumping, and thawing (FPT) as described by Lamarque et al. [61] with some modifications [19]. To this end, 2 g of LMW CHI were mixed with NaOH 50% (w/w) in a solid:liquid ratio of 1:25 (w/v).The heterogeneous mixture was frozen in liquid nitrogen; then the system was pumped under vacuum to eliminate oxygen in order to inhibit CHI degradation in alkaline conditions; and finally thawed at room temperature. The FPT cycle was repeated eight times. Afterwards, the oxygen-free heterogeneous mixture of chitosan with NaOH 50% (w/w) was heated at 120 °C for 2 h in a silicon oil bath to perform the deacetylation reaction. Thereafter, the reaction was stopped and cooled using a mixture bath of acetone and liquid nitrogen. The suspension was neutralized with 4 M HCl till achieving neutrality. Finally, the suspension was centrifuged at 4500 rpm for 30 min using a benchtop centrifuge Hettich ROTINA 420 (Labexchange GmbH, Burladingen, Germany), and the sedimented chitosan powder was washed several times for complete elimination of the salts produced in the neutralization step.

### 4.1. Chitosan Degree of Acetylation (DA)

The CHIs degree of acetylation (DA) were determined by ^1^H nuclear magnetic resonance (^1^H NMR) spectroscopy by the method of Hirai et al. [62]. The ^1^H NMR measurements were performed using a Bruker Avance 250 NMR system at 298 K. The DA was calculated from the ratio of the methyl (CH3) proton signal of the (1→4)-2-acetamido-2-deoxy-β-d-glucan residues to the whole H2 to H6 proton signals as following (Equation (1)),

(1) $$DA\;\left(\%\right)=\frac{\frac{1}{3}\;I_{\left(CH3\right)}}{\frac{1}{6}\;I_{\left(H2-H6\right)}}*100$$

where *I*_(*CH*3)_ is the integral of the CH3 signal at δ ~ 2 ppm, and *I*_(*H*2–*H*6)_ corresponds to the summation of integrals of the signal of the protons H2, H3, H4, H5, H6 and H6′ in the δ range of 3–4.2 ppm.

### 4.2. Chitosan Molecular Weight

The CHI molecular weight was determined by size exclusion chromatography (SEC) coupled to multi-angle laser light scattering (MALLS). Chitosan solutions at 0.1% (w/v) were prepared in an acetic acid/ammonium acetate buffer pH = 4.5 (AcOH (0.2 M)/AcONH4 (0.15 M)), which was used as eluent. Then, they were filtered through 0.45 μm pore size membranes (Millipore, Merck KGaA, Darmstadt, Germany). The chromatographic equipment was composed of an IsoChrom LC pump (Spectra-Physics, Santa Clara, CA, USA) connected to a Protein Pack 200 SW (Waters GmbH, Eschborn, Germany) column and a TSK gel G6000 PWXL. A multiangle laser light scattering detector DAWN DSP (Wyatt Technology Europe GmbH, Dernbach, Germany) operating at 632.8 nm was coupled online to a WATERS 410 differential refractometer.

### 4.3. Thermogravimetric Analysis TGA

The thermal behavior of LMW CHI and the deacetylated-LMW CHI, obtained as above, were analyzed using NETZSCH STA 449 F5 TGA (NETZSCH-Gerätebau GmbH, Selb, Germany). 3–4 mg of chitosan sample were heated from 25 till 750 °C at a heating rate of 10 °C/min under nitrogen gas flow at a rate of 40 mL/min.

### 4.4. Chitosan/Caseinate Interaction in Casted Films

Chitosan–caseinate interactions were first studied by the formation of films from a mixture of the CHI polyelectrolyte solution and the NaCas submicellar dispersion system. Thin films with high molecular weight chitosan HMW CHI of low DA (3%) were prepared, by casting solutions in which chitosan at 1.5% (w/v) was solubilized in water containing stoichiometric amounts of acetic acid as to protonate the chitosan amine groups. For films of chitosan alone, three drying conditions were explored: (1) drying at environmental conditions at room temperature; (2) drying in oven at 40 °C under vacuum (film dried much faster but the rapid drying under vacuum led to formation of bubbles in the film); (3) drying in oven at 40 °C without vacuum (fast drying and, in the film, no bubbles inhomogeneities were observed). Thus, chitosan/caseinate films were obtained by drying in oven (without vacuum) at 40 °C overnight. The chitosan/caseinate films were obtained by mixing HMW CHI solution with sodium caseinate (NaCas) micellar dispersion systems at a NaCas concentration of 1%, 2%, 5%, and 10% in relation to CHI weight.

#### Fourier Transform Infrared FTIR and Raman Spectroscopies

Chitosan–caseinate films and nanoparticles (hydrogel or dried state) were characterized by FTIR in attenuated total reflectance (ATR) mode using a FTIR-ATR Spectrum 65 spectrometer (PerkinElmer, Baesweiler, Germany). Spectra in the range of 400–4000 cm^−1^ were recorded by the accumulation of 64 scans.

Chitosan–caseinate films were also analyzed by Raman Spectroscopy with a BaySpec’s NIR 1064 nm Raman system (RamSpec1064, PhotonLines SAS, Saint Germain-en-Laye, France), at a laser power of 450 mW and exposure time of 20 s.

### 4.5. Preparation of Chitosan-Caseinate Nanoparticles

Chitosan powder was dissolved in a weakly acidic aqueous solution containing a stoichiometric amount of acetic acid to protonate the free amino groups of glucosamine units of CHI chain, to yield a CHI concentration of 0.1% (w/w). Similarly, a dispersion system of 0.1% (w/w) sodium caseinate (NaCas) was prepared by dissolving NaCas powder in distilled water under stirring. Both chitosan solution and caseinate submicellar dispersion were filtered through 0.45 µm pore size membrane (Whatman GmbH, Dassel, Germany) before use.

To obtain CHI–caseinate polyelectrolyte complex (PEC) colloidal systems, the CHI (polycation) solution and NaCas (polyanion) micellar dispersion at the concentration of 0.1% (w/w) were mixed using different volumetric ratios, keeping either CHI or NaCas in excess, and changing the order of addition: chitosan solution into caseinate or vice versa. The volume of the excess solution was kept fixed at 5.00 mL, while the volume of solution being added was varied as 0.10, 0.20, 0.50, 0.75, 1.00, 2.50, and 5.00 mL, respectively. The solutions were allowed to mix for 60 min while magnetically stirring at relatively high speed (ca. 1000 rpm) for few minutes at the beginning, and afterwards the speed was reduced to the half to allow stabilization of the PEC particles. In some experiments, glutaraldehyde (GLU) was added as crosslinker to further promote the hardening of the dispersed particles and verify the formation of distinct nanoparticles in the achieved colloids. To this end, 5 µL of 50% (w/w) GLU were added to the dispersion. The mixture was further stirred for 30 min at low speed to promote formation of nanoparticles and ensure strong binding between CHI and caseinate polymers.

After polyelectrolyte association, PEC nanoparticles could be centrifuged at 2000 rpm for 20 min. From each of the achieved PEC system, 3.50 mL of the supernatant was separately collected for which the zeta potential was further determined. The obtained centrifugate was washed several times to get rid of excess free polymer i.e., not participating in the PEC formation. The sedimented particles were collected for morphological characterizations and drug release experiments. Six different scenarios splitted in Case I and Case II conditions were considered by combining chitosans of different molecular weights (Mw), degrees of acetylation (DA), CHI:NaCas volumetric ratios, and the order of addition of polymer solutions to form the colloidal systems.

Case I: Chitosan in Excess

In each experiment a given volume (0.10; 0.50; 1.00; 2.50, or 5.00 mL) of 0.1% (w/w) sodium caseinate aqueous solution was added one shot at a time to 5.00 mL of 0.1% (w/w) aqueous solution of chitosan of varied Mw and DA as follows:

(i) NaCas added to high molecular weight and low DA chitosan (HMW CHI)
(ii) NaCas added to low molecular weight chitosan with low DA (deacetylated-LMW CHI)
(iii) NaCas added to low molecular weight chitosan with high DA (LMW CHI)

Case II: Sodium Caseinate in Excess

To 5.00 mL of 0.1% (w/w) sodium caseinate (polyanion in excess) aqueous solution, a given volume (0.10, 0.20, 0.50, 0.75, 1.00, 2.50, or 5.00 mL) of 0.1% (w/w) aqueous solution of chitosan of varied Mw and DA was added one shot as follows:

(iv) High molecular weight chitosan (HMW CHI) of low DA added to NaCas
(v) Low molecular weight chitosan of low DA (deacetylated-LMW CHI) added to NaCas
(vi) Low molecular weight chitosan (LMW CHI) of high DA added to NaCas

Table 2 summarizes the different experiments considered in the above described processes: Cases I and II.

### 4.6. Characterization of Chitosan-Caseinate Polyelectrolyte Complexes

#### 4.6.1. Zeta Potential and Hydrodynamic Radius

The chitosan–caseinate polyelectrolyte complex (PEC) hydrodynamic size, zeta potential, size polydispersity index, and electrophoretic mobility were measured using a Malvern Zetasizer Nano ZS (Malvern Panalytical GmbH, Kassel, Germany), equipped with a 4 MW laser operating at wavelength of 633 nm. For each measurement the capillary cell was filled with approximately 1 mL of chitosan solution, caseinate micellar dispersion, or chitosan–caseinate PEC colloidal system. A total of three readings were taken for each sample.

#### 4.6.2. Morphology of Chitosan-Caseinate Nanoparticles

The size, shape, and surface structure of nanoparticles were characterized using transmission electron microscopy (TEM). The centrifugate of the chitosan–caseinate PEC system was washed several times with distilled water. Thereafter a droplet containing the washed chitosan–caseinate centrifugate (5 µL) was deposited on carbon coated copper grids (CF400-CU Carbon Film, 400 Mesh Copper grids) which were previously cleaned with plasma to get rid of surface contamination and make the grid more adhesive towards the sample, and allowed to dry for 1 min. Excess solution was wiped off and the sample was negatively stained with 3 µL of uranyl acetate for 2 min. The samples were observed with TEM microscope, model Zeiss LEO 912 Omega (Carl Zeiss Microscopy GmbH, Jena, Germany).

### 4.7. Insulin-Loaded Chitosan-Caseinate Nanoparticles for Drug Controlled Release

Insulin, which is a hormone used in the treatment of diabetes as it is responsible for the cell uptake of glucose, was used as model to explore potential application of the chitosan-caseinate nanoparticles (NPs) as carriers in drug-controlled release. The NPs chosen for this study were those obtained when 2.50 mL of 0.1% (w/w) high molecular weight chitosan (HMW CHI) of low DA = 3% were added to 5.00 mL of 0.1% (w/w) caseinate. To prepare the insulin entrapped chitosan–caseinate nanoparticles, two models were envisioned as described above in Scheme 1. 2.7 mg of insulin were added either to the HMW CHI solution (0.1% (w/w), 2.50 mL) prior to its addition to the NaCas in excess (Model I), or to the NaCas (polyelectrolyte in excess) to which 2.50 mL of 0.1% (w/w) HMW CHI were added afterwards (Model II). Insulin was allowed to mix slowly with HMW CHI or NaCas, respectively, and stirred for about 15 min. In the case of association Model I, the insulin/HMW CHI solution was added to NaCas solution, dropwise. In the case of association Model II, the HMW CHI solution was added to the insulin/NaCas solution dropwise. The final insulin/chitosan/caseinate dispersions were magnetically homogenized for 60 min, after which they were centrifuged at 14500 rpm for 30 min. The centrifugate allowed recovering the insulin-entrapped chitosan–caseinate nanoparticles. The supernatant was carefully removed and used to determine the amount of non-entrapped/free insulin by UV spectrometry.

#### Insulin Entrapment Efficiency and Release Kinetics. UV Spectroscopy

The insulin entrapment efficiency (EE) in the chitosan–caseinate nanoparticles was determined using a Lambda 35 ultraviolet visible (UV-Vis) spectrophotometer (PerkinElmer, Baesweiler, Germany). To build the insulin concentration calibration curve, the UV spectrum of different insulin standard solutions was analyzed. The absorbance peak at the wavelength of 276 nm related to insulin, determined for the different insulin concentrations, was plot in the calibration curve. This was used to determine by UV spectroscopy the concentration of free insulin in the supernatant after centrifugation of the processed insulin-loaded chitosan–caseinate nanoparticles. Then, the insulin entrapment efficiency was estimated using Equation (2):

(2) $$\begin{array}{c} Entrapment\;efficiency\;\left(\%\right)\\ \;=\frac{Total\;amount\;of\;insulin-Amount\;of\;free\;insulin}{Total\;amount\;of\;insulin}\times 100 \end{array}$$

where free insulin corresponds to the insulin measured in the supernatant fraction.

To study the kinetics of insulin release, the insulin-entrapped chitosan–caseinate nanoparticles collected after centrifugation were added to 3 mL of phosphate-buffered saline (PBS) (pH = 7.4), which simulated the environment of human intestine and bloodstream both having pH around 7.0 to 7.5. The whole system was submerged in a water bath maintained at 37 °C and allowing for horizontal shaking at 75 rpm. After a given time, the sample was centrifuged again at 14,500 rpm for 15 min. Then, small aliquot from the supernatant was removed for evaluation of the insulin percentage release Pt at the given time t. The same aliquot volume of PBS was resupplied to the supernatant which was again mixed with the insulin-entrapped chitosan–caseinate nanoparticles. This procedure was repeated for the times 1, 2, 14, 18, and 24 h. The concentration of released insulin was determined by UV spectrometry by using the insulin calibration curve obtained as explained above. The cumulative percentage of drug release was determined following Equation (3):

(3) $$Cumulative\;percent\;release=\frac{Volume\;of\;aliquot\;withdraw}{Bath\;volume}\times P_{\left(t-1\right)}+P_{t}$$

where, *P_t_* = percentage release at time t, *P*_(*t*−1)_ = percentage release prior to time t.

## 5. Conclusions

In this work, fully natural and biodegradable polyelectrolyte complex (PEC) colloidal nanoparticles of chitosan (CHI) and caseinate were prepared, and the structural and electrostatic features of these PEC colloids were systematically studied, which led to the reproducible formation of CHI–caseinate nanoparticles (NPs) with diameter ranging from 15 to 200 nm in the dried state, and hydrodynamic radius around 200 nm for colloidal hydrogels. Several parameters of the processing and polyelectrolyte nature of the used biopolymers have influenced on the characteristics of the obtained NPs in terms of zeta potential, hydrodynamic radius, and association topology. The process of particle formation depended on the polymer being taken in excess (CHI or caseinate), the added volume of polymer in defect and the chitosan macromolecular structure, especially on the CHI degree of acetylation (DA) with the determining positive role of a low DA. Then, sodium caseinate is known for its self-assembly association which organizes as sub-micelles. Thus, the final structure of CHI–caseinate nanoparticles resulted from the existence of preformed sodium caseinate sub-micelles on which chitosan can bind by electrostatic complexation and stabilize the colloids, keeping a positive charge resulting from an incomplete chitosan neutralization. The NaCas sub-micellar nanosubstrate offers a large interaction surface to chitosan chains in solution available for PEC formation and stabilization.

Finally, the potential use of the chitosan–caseinate nanoparticles as nanocarriers for drug delivery was assessed by using insulin as model drug. The insulin entrapment efficiencies in the chitosan–caseinate nanoparticles was higher than 75% in the different proposed chitosan/caseinate/insulin association models. The interaction between insulin, chitosan, and caseinate should be considered altogether to understand the competitive effects in the establishment of macromolecules interactions. Our results confirm the preferential binding of insulin to highly deacetylated chitosan in the most outer part of the particles (Model I). Competitive binding of caseinate and insulin to chitosan in the inner particle regions (Model II) seems to also play a role in the controlled-release of insulin.

## Figures and Tables

**Table 1:** Macromolecular structure parameters of the different chitosans.

Type of Chitosan	Mn (g/mol)	Mw (g/mol)	PDI	DA (%)
HMW CHI	4.10 × 10^5^ ± 6.4%	6.11 × 10^5^ ± 9.6%	1.49 ± 11.6%	3
LMW CHI	3.87 × 10^5^ ± 0.62%	5.23 × 10^5^ ± 0.73%	1.34 ± 0.35%	13
deacetylated-LMW CHI	7.52 × 10^4^ ± 0.59%	1.75 × 10^5^ ± 0.31%	2.327 ± 0.67%	3

number (Mn) and weight-average molecular weight (Mw), degree of acetylation (DA), polydispersity index (PDI).

**Table 2:** Summary of the different formulations considered by varying processing parameters in the different experiments of the Cases I and II.

Experiment	Chitosan Mw	Chitosan DA	Volume Ratio V(CHI):V(NaCas)
**Case I: Chitosan in excess**			
(i)	High	Low	50:1; - ; 10:1; - ; 5:1; 2:1; 1:1
(ii)	Low	Low	50:1; - ; 10:1; - ; 5:1; 2:1; 1:1
(iii)	Low	High	50:1; - ; 10:1; - ; 5:1; 2:1; 1:1
**Case II: Caseinate in excess**			
(iv)	High	Low	1:50; 1:25; 1:10; 3:20; 1:5; 1:2; 1:1
(v)	Low	Low	1:50; 1:25; 1:10; 3:20; 1:5; 1:2; 1:1
(vi)	Low	High	1:50; 1:25; 1:10; 3:20; 1:5; 1:2; 1:1
